# Growth variation of an ambrosia fungus on different tree species indicates host specialization

**DOI:** 10.3389/finsc.2025.1696497

**Published:** 2025-12-16

**Authors:** Marcel Hugo Decker, Peter H. W. Biedermann, Lennart J. J. van de Peppel, Jon Andreja Nuotclà

**Affiliations:** Albert-Ludwigs-Universität Freiburg, Chair of Forest Entomology and Forest Protection, Stegen-Wittental, Germany

**Keywords:** ambrosia fungus, *Dryadomyces montetyi*, ambrosia beetle, mutualism, host specialization, *Platypus cylindrus*

## Abstract

Ambrosia beetles rely on mutualistic fungi as a food source for themselves and especially for their offspring, yet the influence of host tree species on fungal growth and specialization remains poorly understood. In this study, we investigated the growth performance of the ambrosia fungus *Dryadomyces montetyi*, an important nutritional symbiont of the oak pinhole borer *Platypus cylindrus*, on semi-artificial media infused with extracts of four tree species: *Quercus robur*, *Fagus sylvatica*, *Abies alba*, and *Pseudotsuga menziesii*. Fungal growth was quantified over time using logistic models of the growth area and final dry weight measurements. The growth of *D. montetyi* differed significantly among the different host tree substrates. Growth on *F. sylvatica* was comparable to that on *Q. robur*; however, both conifer-derived media (*A. alba* and *P. menziesii*) exhibited significantly reduced surface expansion speed. Interestingly, growth speed on the European native *A. alba* was measurably higher than on the non-native *P. menziesii. Q. robur* medium had the highest fungal density of all tree hosts. However, density estimates were close and only nutrient-rich laboratory growth medium without tree extract differed significantly, as it had by far the highest density as well as growth speed of all measured media. Our findings show that fungal performance reflects the known preference of *P. cylindrus* for deciduous host trees. Host-related specialization of the fungal symbiont certainly determines host tree selection by *P. cylindrus*, which affects the evolution of the tripartite interactions between beetle, fungus and host trees.

## Introduction

Ambrosia beetles are not a taxonomic classification but rather a polyphyletic group of beetles that share fungus farming as a common ecological lifestyle (1). Fungus farming evolved several times independently in the weevil subfamilies Scolytinae, once in Platypodinae (2), and once in the superfamily Lymexyloidea (3). Ambrosia beetles maintain an obligate mutualistic association with so-called ambrosia fungi, whose spores they carry in highly specialized organs (mycetangia; 4) and inoculate into the wood while excavating breeding tunnels (5–7). Both larvae and adults obligately rely on these fungi for nutrition (7). Inside their galleries, ambrosia beetles actively manage their fungal symbionts and prevent the spread of competing microorganisms (6, 8–11). Most ambrosia fungi are members of the phylum Ascomycota, particularly within the orders Ophiostomatales and Microascales and occur in genera such as *Ambrosiella*, *Raffaelea*, *Dryadomyces* and *Ophiostoma* (12, 13). Today, many ambrosia fungi rely entirely on beetles for survival, whereas their ancestors lived freely and spread with the help of arthropods (14, 15).

The ambrosia beetle *Platypus cylindrus* (Fabricius, 1792; Coleoptera: Curculionidae: Platypodinae), also known as the oak pinhole borer (16), belongs to the Coleopteran subfamily Platypodinae (17), which comprises more than 1,400 described species. The majority of these species are native to tropical regions, and with only two known exceptions, all are ambrosia beetles (18). Next to *P. cylindrus*, only one other platypodine species occurs in southern Europe: *Platypus oxyurus* Dufour. It occurs in the Pyrenees, Turkey, Corsica, Calabria, and Greece and is strictly associated with silver fir (*Abies alba* Mill.) (19). A *Graphium* sp. has been isolated from *P. oxyurus* (20), but it is unclear if it is its main symbiont, because typically Platypodinae are associated with *Raffaelea* or *Dryadomyces* spp. (17). In contrast*, P. cylindrus* has been recorded on various broadleaf tree species. The principal hosts are *Quercus* spp., along with *Fagus sylvatica* L. (21). Additional host species include *Castanea sativa* L. (22), *Ulmus* spp. (23), *Prunus avium* L. (20), *Juglans regia* L. and *Fraxinus excelsior* L. (24). This diversity demonstrates the wide potential host range of *P. cylindrus* among deciduous tree species, even though oak species are preferred (21). This relatively polyphagous nature of *P. cylindrus* should be linked to the polyphagy of its symbiotic fungi, but that has not been tested. More generally, polyphagy is an ancestral trait and possibly an evolutionary adaptation to the high floristic diversity of tropical ecosystems, where most ambrosia beetle taxa originate. In such ecosystems, narrow tree host specialization would be disadvantageous for both beetles and their fungal mutualists (25, 26).

The ecology of *P. cylindrus* has been extensively studied before the mid-20^th^ century (16, 21, 23, 27–29; reviewed in 30), while recent studies have focused on the fungi associated with *P. cylindrus* (16, 31–33). However, platypodine ambrosia beetles are typically associated with a richer fungal community than other ambrosia beetle groups (e.g. compared to *Xylosandrus* ambrosia beetles; 14), so the role and importance of individual microbial species for the beetles is not fully clear. The currently increasing economic relevance of *P. cylindrus* calls for a good understanding of the associated microbial players (30, 34).

An early study reported *Raffaelea ambrosiae* Arx & Hennebert as the nutritional symbiont of *P. cylindrus* from Great Britain (16, 35). More recent studies from Portugal and Algeria also report *Dryadomyces montetyi* (M. Morelet) M. Procter & Z.W. de Beer (synonym *Raffaelea montetyi* M. Morelet) and *Raffaelea canadensis* L. R. Batra as primary symbionts (31–33). However, while *P. oxyurus* shows a strict host preference for silver fir, *P. cylindrus* has been observed on several broadleaved tree species, indicating a specialization on broadleaves rather than on a single host. This strong host preference may be attributed to differences in the establishment success of their fungal symbionts. For successful colonization, nutritional fungi must quickly establish within freshly excavated tunnels and maintain a competitive advantage over other microbial taxa.

Experimental studies have shown that ethanol can act as a selective factor, enhancing the initial growth and competitive success of ambrosia fungi after breeding tunnels are inoculated with their spores (15, 36). Furthermore, optimal temperature conditions (37) and the concentration of key nutrients such as nitrogen (38) significantly affect fungal growth. While these factors have been extensively investigated, the role of the host tree species in shaping the growth performance of ambrosia fungi remains poorly understood. We hypothesize that the host tree species significantly affects the growth of *D. montetyi*, because of the tree-host preference of its beetle associate *P. cylindrus*, which may reflect patterns of host-tree specialization by the fungus. This would imply that co-evolution may have occurred not only between this beetle and its main fungal symbiont, but also between *D. montetyi* and oak trees. To test this hypothesis, we measured the performance of *D. montetyi* on semi-artificial growth medium infused with wood extracts from four different tree species.

## Materials and methods

### Fungus strain

For all our experiments we used a single strain of *D. montetyi* (F80052) from our inhouse culture collection at the Chair of Forest Entomology and Forest Protection in Stegen, Germany. This strain was isolated from *P. cylindrus* infesting an oak stump at a forest edge east of Freiburg, Germany (360 m a.s.l.) in June 2023 (see Appendix for detailed methods). A partial sequence of the ribosomal 28S large subunit (LSU) sequence of this strain is available on NCBI GenBank under the accession number PX240738.

### Fungal growth on different modified wood substrates

**Figure 1:**
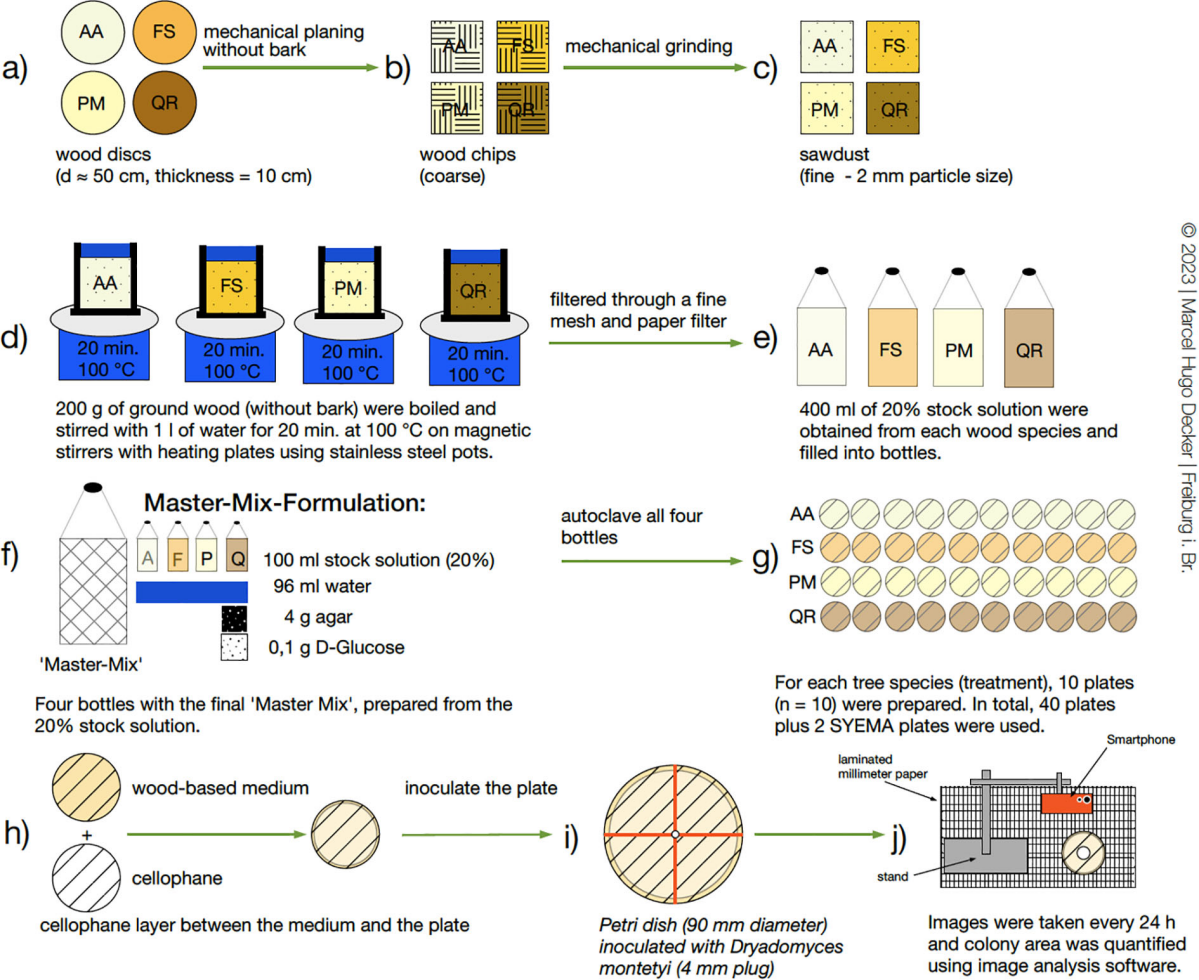
Preparation of wood-extract “Master-Mix” medium and plate assay. Wood from four host trees was processed into sawdust, extracted to 20% stocks, combined into a standardized agar medium, poured, and centrally inoculated with *D. montetyi* for growth and biomass measurements. AA, *Abies alba*; FS, *Fagus sylvatica*; PM, *Pseudotsuga menziesii*; QR, *Quercus robur*.

Wood discs of *Abies alba* Mill., *Pseudotsuga menziesii* (Mirbel) Franco, *Fagus sylvatica* L., and *Quercus robur* L. (48–56 cm diameter, ~10 cm thickness) were obtained from local sawmills. After five days of storage at room temperature (25 °C), the discs were planed and the resulting wood chips subsequently ground with a rotor mill (18,000 rpm, 2 mm sieve). From 200 g sawdust per tree species, a 20% stock solution was prepared by boiling it in 1 L water for 20 min at 100 °C. It was then filtered and further processed into a standardized Master-Mix medium (see **Figure 1**). After autoclaving, the medium was poured into Petri dishes (90 mm × 15 mm; 20 ml per dish).

Each plate was lined with a cellophane layer and inoculated with a mycelium plug of 2 mm radius taken from a 10-day-old *D. montetyi* culture grown on nutrient-rich SYEMA medium (3 g yeast extract, 10 g malt extract, 15 g agar, 100 mg Streptomycin, 1 L water). Note that streptomycin is principally not needed for the current experiment but was retained to follow our usual laboratory protocol where it helps reduce bacterial contamination risk. Inoculation was performed centrally using a punch tool, with millimeter paper as positional reference. In total, 40 plates were prepared for the main experiment (10 replicates per tree species) plus two additional SYEMA plates as high-nutrient reference. Fungal growth was documented every 24 h under sterile conditions by orthogonal photographs and quantified with ImageJ (version 1.53k) until either the surface of the Petri dish was fully covered, or four days had elapsed. Subsequently, the mycelium was scraped off the cellophane, dried for 5 days at 50 °C and weighed with a precision balance (Kern PNJ, accuracy 1 mg). Biomass was calculated by differential weighing of empty versus mycelium-containing micro reaction tubes.

### Statistical analysis

First, we characterized the growth of fungal propagules over time fitting a logistic growth model to the measured fungus area data: $Area_{day}=\frac{A}{1\;+\;e^{-\left(day-C\right)/B}}$

A is the asymptotic maximum area, thus the theoretical maximum growth area

B is the time scaling parameter, indicating growth speed

C is the inflection point, thus the timepoint with fastest fungal growth

Peak growth (= absolute growth at the inflection point) has a more intuitive biological meaning than *B* and was thus calculated from the derivative ${\frac{\text{d}A}{\text{d}t}|}_{C}=\frac{A}{4\cdot B}$.

Logistic models were fitted individually for each plate using nlsList in the statistics software R, allowing for plate-specific parameter estimates (see **Supplementary Figure 1** for individual curve fits). Standard errors (SE) were extracted to account for differences in measurement precision. We subsequently fitted weighted linear models for peak growth and the logistic growth parameters *A* and *C* to assess fungal growth across media derived from different host tree species. Each model had the form: $Y_{i}=\beta_{0}+\beta_{1}species+\beta_{2}species\ldots +\epsilon_{i},\;weights=\frac{1}{SE_{i}^{2}}$, where $Y_{i}$ is the parameter of interest, $\beta_{0}$ represents growth medium derived from *Q. robur* as the reference, and $\beta_{j}$ are differences between medium obtained from *Q. robur* and each of the other three tree species as well as complete nutrient-rich SYEMA medium. *Post-hoc* pairwise comparisons were not performed, as *Q. robur* is the main host and provides the biologically relevant baseline.

Finally, we calculated the fungal tissue density for each plate by dividing the dry weight by the total growth area on the last day of measurement. Those values were then also compared between the different growth media using linear models in the statistical software R. The *P. menziesii* treatment was excluded from this analysis because the mycelial mass was too low to be accurately measured with our method.

All analyses were conducted in Rstudio (39) with R version 4.4.2 (40), using the nlme (41, 42), dplyr (43), ggplot2 (44), writexl (45) and ggbreak (46) packages.

## Results

**Figure 2:**
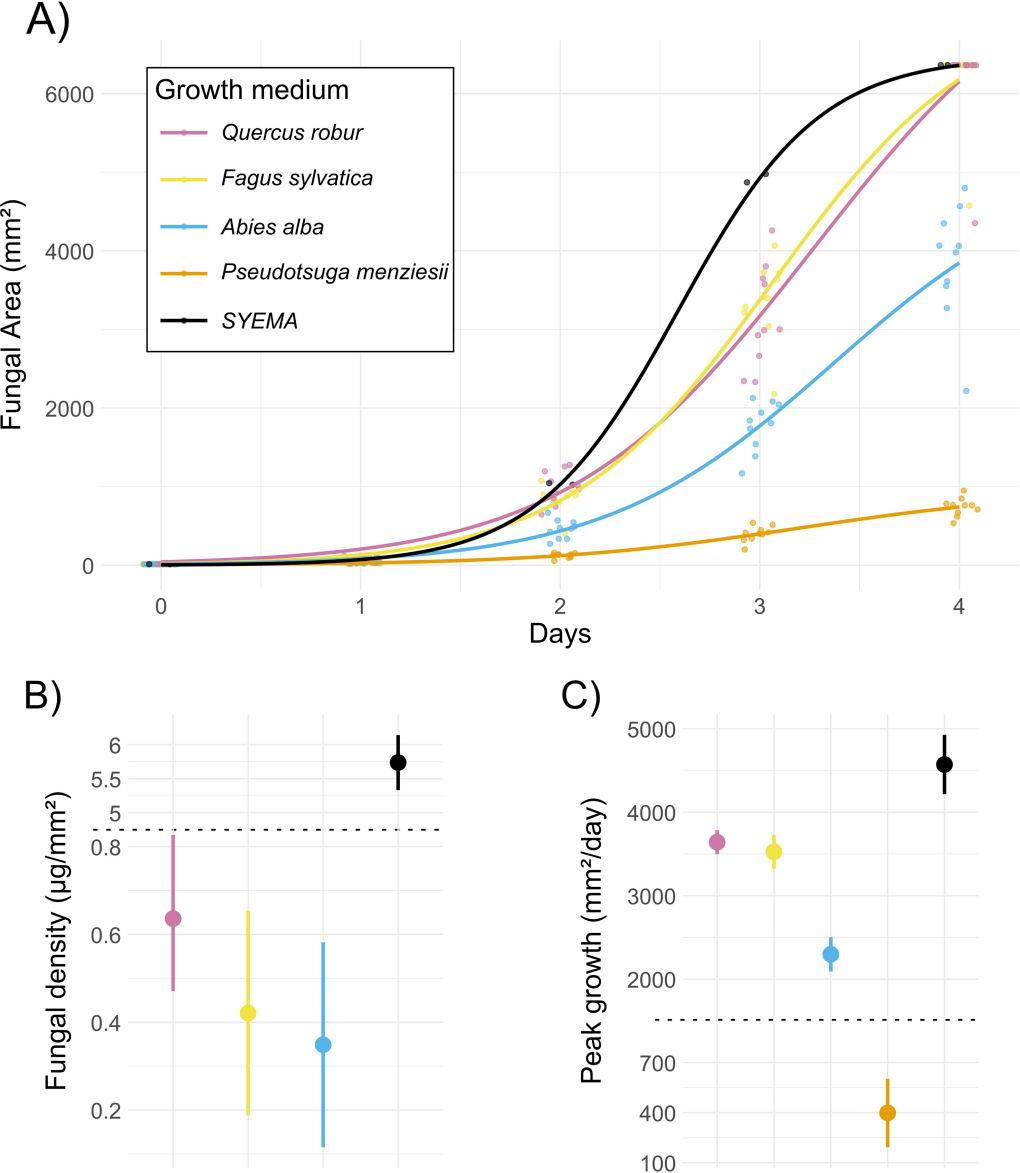
**(A)** Logistic growth curves of fungal colony expansion on different growth media. Growth media were either infused with extracts from different tree species (see **Figure 1**) or with an antibiotic and a high concentration of sugar and nutrients in the case of the standard laboratory growth medium Streptomycin–Yeast Extract–Malt Agar (SYEMA)- Yeast extract- Malt- Agar (SYEMA). Dots represent observed colony areas for replicate plates, with small horizontal jitter to reduce overlap, while solid lines show species-level logistic fits. **(B)** Fungal density across media, shown as estimated means with error bars indicating standard errors. A pseudo-broken y-axis compresses the high values of SYEMA to allow clearer visualization of variation among the other media (y-axis break at the dotted line). **(C)** Peak daily growth rates across media, shown as estimated means with standard errors. A pseudo-broken y-axis compresses the extreme values, enabling comparison of lower rates across species (y-axis break at the dotted line).

Fungal growth and biomass differed significantly among growth media, with *Q. robur* growth medium serving as a biologically relevant reference. The calculated maximum area (*A*) was highest on *Q. robur* (6888 mm²), and significantly lower on *A. alba* (by −2838 mm², p < 0.001) and *P. menziesii* medium (by −5953 mm², p < 0.001), whereas *F. sylvatica* (by −703 mm², *p* = 0.104) and the complete *SYEMA medium* (by −411 mm², p = 0.30) did not differ significantly from the reference (**Table 1**, **Figure 2**, see **Supplementary Table 1** in the Supplementary Material for detailed model outputs).

**Table 1:** Mean growth parameters of the ambrosia fungus *D. montetyi* across different test media.

Grow medium	Max area (*A*, mm²)	Inflection point (*C*, days)	Peak growth (mm²/day)	Density (μg/mm²)
*Quercus robur*	6888	2.933	3641	0.636
*Fagus sylvatica*	6185	3.022	3526	0.421
*Abies alba*	4050	3.129	2297	0.349
*Pseudotsuga menziesii*	934	3.045	398	–
SYEMA (lab substrate)	6477	2.589	4571	5.737

Shown are the mean maximum colony area (A, mm²), the mean inflection point of the growth curve (C, days), the mean peak growth rate (A/4B, mm²/day) at the inflection point, and the final fungus density (µg/mm²).

The inflection point (*C*) occurred after 2.93 days or 70 hours on *Q. robur* medium, whereas fungus on the complete *SYEMA* medium reached peak growth earlier (by −0.344 days or –8 hours, p < 0.001) and *A. alba* medium slightly later (by +0.196 days or +5 hours, p = 0.050). C did not differ significantly between *Q. robur* and *F. sylvatica* or *P. menziesii* respectively (**Table 1**, **Figure 2**, **Supplementary Table 1**).

Peak growth rate was 3641 mm^2^ per day in *Q. robur* and was not significantly different in *F. sylvatica* medium (by +115 mm^2^ per day, p = 0.579). Both, media sourced from *A. alba* (by −1344 mm^2^ per day, p < 0 .001) and *P. menziesii* (by −3243 mm^2^ per day, p < 0.001) had significantly lower peak fungal growth rates, however, on complete SYEMA medium, the peak growth rate was significantly higher than in *Q. robur* (by +931 mm^2^ per day, p = 0.013; **Table 1**, **Figure 2**, **Supplementary Table 1**).

Tissue density was 0.636 μg/mm² on *Q. robur* medium and significantly higher on complete *SYEMA* medium (by +5.101 μg/mm², p < 0.001). We found no statistically significant fungal tissue density difference between *Q. robur* and both, *F. sylvatica* and *A. alba* respectively (**Table 1**, **Figure 2**, **Supplementary Table 1**).

## Discussion

In summary, among all tree-infused media, the ambrosia fungus *D. montetyi* grew faster on medium derived from the deciduous *Q. robur* wood than on that derived from the two conifer species. We found no difference in fungal growth between the two deciduous tree-derived media. Although a full set of multiple comparisons was not appropriate for this study design, *D. montetyi* showed markedly faster growth on the medium prepared from the European native conifer *A. alba* than on that from the non-native *P. menziesii*. As expected, the nutrient-rich standard laboratory medium SYEMA promoted both significantly higher growth rates and a much denser fungal tissue.

### Methodology

We demonstrate that growing *D. montetyi* on a semi-artificial medium composed of wood extract and agar is an effective method to differentiate fungal performance on different host tree species, as we could clearly observe significant differences in radial growth parameters and mycelial density between treatments. Here we were limited to using a single strain of *D. montetyi*, but as ambrosia fungi generally exhibit low intraspecific variation (47), we expect that strain-specific differences play only a minor role. However, further studies using multiple strains are needed to confirm this. We also show that radial growth or fungal surface area alone is not sufficient to measure fungal performance, as two colonies with a similar final growth area, like *Q. robur* and the nutrient-rich SYEMA medium can produce mycelium that strongly differs in its density. Although we can recommend density calculated from dry mass and final growth area as a reliable metric (see e.g. 36), some caution is advised. The mycelial mass is generally only in the order of a few milligrams when fully covering standard sized petri dishes. Thus, very high precision measurements are needed to accurately measure slow growing fungi. In our study, we had to discard the measurements for the *P. menziesii* medium, as the fungal growth was below the detection threshold of our one-milligram precision scale. Using higher precision scales or spectrometry approaches could solve this issue. However, the amount of biomass produced on the other types of media was also low, but measurable, as the fungus covered almost the entire plate after three to four days. Thus, to obtain sufficient fungal biomass for comparing treatments with slow growing fungi, we recommend future studies to extend the duration of the experiment and use larger petri dishes or race tubes (e.g. 48). This allows prolonged fungal growth and a better comparison of growth characteristics among the treatments with faster growing fungi. Finally, it may be worth testing qPCR approaches measuring fungal DNA or quantifying nutrients, for example, ergosterol levels as alternative proxies (49).

We show that *D. montetyi* performs significantly better on the deciduous host tree species that are preferred by the host beetle *P. cylindrus*. Not only does it grow faster on *Q. robur* and *F. sylvatica* medium in comparison to the medium containing extracts of the two conifer species, but it covers significantly larger areas before the growth speed declines. Additionally, it did produce denser mycelium on *Q. robur* relative to the others, although these differences were not statistically significant (see **Figure 2**).

Our experiment is able to show differences in fungal growth but cannot explain the cause of these differences. The reduced fungal growth on *A. alba* and even more strongly on the *P. menziesii* medium could be because of inhibition caused by defensive chemicals produced by the host tree, differences in sugar concentration or the lack of certain essential micronutrients. To find an explanation for the observed differences in fungal growth between the different treatments, future studies need to analyze the wood extracts and determine the concentration of macro and micronutrients and tree defensive chemicals before and after the fungus has grown on the plates. Polysaccharides such as cellulose and hemicellulose, which are the main structural components of wood, are relatively stable at temperatures below 180 °C (50). However, some minor heat-sensitive compounds that are important under natural conditions may have degraded during sterilization.

### Tree host specialization of *D. montetyi*

Interestingly, *D. montetyi* is not strictly associated with *P. cylindrus*, but has also been found in association with *Xyleborus dryographus* (Ratzeburg, 1837) and *Xyleborus monographus* (Fabricius, 1793) that also inhabit oak trunks (51). The *D. montetyi* strains associated with these three different beetle species do not show species-specific genetic variation, indicating recent and potentially ongoing horizontal transfer. Horizontal transfer between these beetle species has been attributed to their shared use of oak trunks and to the fungus’s ability to invade neighboring galleries through woody tissue, a process which should be facilitated by its faster growth rate relative to other ambrosia fungi (51). An alternative explanation could be joint usage of tunnels as has been recently reported to be quite common in some ambrosia beetle species (52). Occasional horizontal symbiont transfer between *P. cylindrus* and other unrelated ambrosia beetle species may have prevented the evolution of a strong host-symbiont fidelity of *D. montetyi*.

As most ambrosia beetles have a broad range of host trees, we did not expect that their fungi are more selective (25, 26). Our findings do not support a broad potential host range as we observe that *D. montetyi* grows significantly faster on deciduous compared to conifer tree-hosts, indicating that there is at least some degree of specialization by the fungus. Unfortunately, there are very few experimental studies that further tested this hypothesis. Castrillo et al. (53) found significant differences in radial growth of different strains of *Ambrosiella grosmanniae* Mayers, McNew & Harr., the fungal symbiont of *Xylosandrus germanus* (Blandford, 1894), on medium infused with sawdust of different host tree species. However, they only measured radial growth once after two days and did not measure fungal biomass or density. Therefore, it remains unclear whether the case of *D. montetyi* is an exception or whether host tree specialization occurs more frequently.

In the context of forestry, it is good news that the specialization of *D. montetyi* appears to be relatively narrow, which aligns with the observed preference of the beetles for deciduous host trees (21). This is because the economic damage caused to cork oaks in the Mediterranean and oak forests in Central Europe by *P. cylindrus* is currently increasing rapidly (30). While it is highly likely that this is connected to declining tree health as a result of climate change, concerns have been raised that high *P. cylindrus* populations may also affect alternative hosts, such as *F. sylvatica*, as well as proposed future tree species for Central European forests, such as *A. alba* and *P. menziesii*. At least for its fungal symbiont, the all-clear can be given for the two conifer species. More detailed analyses of both *P. cylindrus* and its symbionts are necessary for *F. sylvatica* to clarify how likely and successful host switches may be.

It is not known if host tree specialization of *D. montetyi* also affects host beetle fitness or behavior and thus may select for host tree preference by the beetle. Alternatively, in evolutionary history, specialization by beetle hosts may have resulted in subsequent specialization by the fungus. Some studies have experimentally measured ambrosia beetle fitness on semi-artificial substrates using sawdust of different host tree species and found conflicting results. A study on *Xyleborus glabratus* (Eichhoff, 1877) found lower success rates and decreased productivity on non-preferred host trees (54) and a study on *X. germanus* found significant differences in progeny produced among four different host tree species even though this beetle is regarded to be a host generalist (53). Contrary to this, a study on the host-tree generalist, *Xyleborinus saxesenii* (Ratzeburg, 1837) found no effect of host-tree species on beetle fitness (55).

Our findings may indicate why studies come up with opposing results, as we report significant improvements of fungal growth speed and density for the very nutrient-rich SYEMA vs *Q. robur* medium. This highlights the importance of considering the nutrient content of artificial media that have been used in all the studies above, as it is almost impossible to measure fungal biomass directly on the wood due to the ability of the fungus to penetrate the wood (5, 56). For future studies it would be interesting to test if the differences in fungal growth rate that we observe also align with difference in fitness of *P. cylindrus*. However, attempts to rear *P. cylindrus* on artificial medium showed that it is not as easy as for sib-mating Xyleborini (57).

To understand the complex interactions that lead to host-symbiont fidelity, it is crucial to study all partners involved. Except for a few studies mentioned above, most studies have focused on the host tree preference of the beetle (53–55), but have not considered possible host tree specialization by their symbiotic fungi. Testing both the performance of ambrosia beetles and their fungal symbionts on different tree hosts will help in identifying and understanding the drivers that shape host-fidelity and the evolution of obligate fungus-farming.

## Data Availability

The raw data supporting the conclusions of this article will be made available by the authors, without undue reservation.
